# Evaluating Nutritional Risk Factors for Delirium in Intensive-Care-Unit Patients: Present Insights and Prospects for Future Research

**DOI:** 10.3390/clinpract13060138

**Published:** 2023-12-07

**Authors:** Arianna Piccirillo, Francesco Perri, Alessandro Vittori, Franco Ionna, Francesco Sabbatino, Alessandro Ottaiano, Marco Cascella

**Affiliations:** 1 Otolaryngology and Maxillo-Facial Surgery Unit, Istituto Nazionale Tumori—IRCCS Fondazione G. Pascale, 80131 Naples, Italy; 2 Medical and Experimental Head and Neck Oncology Unit, Istituto Nazionale Tumori—IRCCS Fondazione G. Pascale, 80131 Naples, Italy; 3 Department of Anesthesia and Critical Care, ARCO ROMA, Ospedale Pediatrico Bambino Gesù IRCCS, Piazza S. Onofrio 4, 00165 Rome, Italy; 4 Medical Oncology Department, University of Salerno, 84084 Fisciano, Italy; fsabbatino@unisa.it; 5 SSD Innovative Therapies for Abdominal Metastases, Abdominal Oncology, Istituto Nazionale Tumori di Napoli, IRCCS “G. Pascale”, 80131 Naples, Italy; a.ottaiano@istitutotumori.na.it; 6 Unit of Anesthesiology, Intensive Care Medicine, and Pain Medicine, Department of Medicine, Surgery, and Dentistry, University of Salerno, Via Salvador Allende, 43, 84081 Baronissi, Italy

**Keywords:** malnutrition, intensive care medicine, delirium, hypercatabolism, bioelectrical impedance analysis

## Abstract

Malnutrition, hypercatabolism, and metabolic changes are well-established risk factors for delirium in critically ill patients. Although the exact mechanisms are not fully understood, there is mounting evidence suggesting that malnutrition can cause a variety of changes that contribute to delirium, such as electrolyte imbalances, immune dysfunction, and alterations in drug metabolism. Therefore, a comprehensive metabolic and malnutrition assessment, along with appropriate nutritional support, may help to prevent or ameliorate malnutrition, reduce hypercatabolism, and improve overall physiological function, ultimately lowering the risk of delirium. For this aim, bioelectrical impedance analysis can represent a valuable strategy. Further research into the underlying mechanisms and nutritional risk factors for delirium is crucial to developing more effective prevention strategies. Understanding these processes will allow clinicians to personalize treatment plans for individual patients, leading to improved outcomes and quality of life in the intensive-care-unit survivors.

## 1. Introduction

Delirium is a transient acute neuropsychiatric disorder characterized by the alteration in multiple cognitive functions (mainly attention and executive functions) that occurs following the onset of an acute clinical problem and is the expression of cerebral metabolic suffering [1]. It is a very frequent phenomenon of acute brain dysfunction that affects up to 80% of patients in the intensive care unit (ICU), especially in post-surgical or traumatic contexts [2]. This serious clinical condition is linked to elevated mortality rates, extended hospital stays, greater medical expenses, and long-lasting cognitive impairment [3].

Based on psychomotor behavior, delirium is classified into three subclasses, namely hyperactive, hypoactive, and mixed delirium. Hyperactive delirium is characterized by agitation, hallucinations, and restlessness. The hypoactive subtype is featured by apathy, decreased reactivity, slowed motor function, withdrawn attitude, lethargy, and somnolence [4]. This subtype is associated with a worse prognosis [5]. Mixed delirium is a fluctuation between hypoactive and hyperactive types. This is the most frequent subtype and accounts for about half of the total cases [6,7].

Due to the potentially devastating consequences, including delayed functional recovery, prolonged hospital stays, and higher mortality rates [8,9], careful assessment and treatment of this condition are essential in critical care medicine. Moreover, prevention is a critical component of delirium management as it can help to reduce the incidence and severity of delirium episodes and improve patient outcomes [10]. Notably, while delirium can be treated, its prevention is often more effective than treatment [10]. Therefore, preventive measures must be implemented to identify and minimize the factors that contribute to the development of delirium. In this regard, different pharmacological and non-pharmacological strategies have been investigated to prevent delirium, with mixed results [11,12,13].

Several modifiable and non-modifiable risk factors can contribute to the development of delirium. While non-modifiable risk factors, such as advanced age, pre-existing cognitive impairment, or a history of dementia or other neurological disorders, cannot be changed or controlled, identifying and modifying modifiable risk factors through appropriate interventions can help to reduce the risk of delirium and improve patient outcomes [14]. For example, addressing underlying medical conditions, such as infections or electrolyte imbalances, may reduce the risk of delirium [15].

In this context, malnutrition is recognized as a significant risk factor for delirium, especially in older individuals when in long-term care. Several investigations demonstrated that 75% of hospitalized older patients with delirium also suffered from malnutrition, suggesting a significant association between malnutrition and delirium. In addition, patients with both malnutrition and delirium had a fourfold increase in mortality rate during a one-month follow-up, a seven times higher discharge rate to nursing homes, and a longer hospital stay of three days compared to those without malnutrition or delirium [16,17,18]. Nevertheless, establishing an association between malnutrition and delirium in critically ill patients is a complex task due to the multifactorial genesis of these two phenomena and the concomitant presence of multiple comorbidities and risk factors.

Employing a narrative review of the literature, this manuscript provides an overview of the causes of malnutrition in critically ill patients, the strategies for the assessment of malnutrition, and the underlying mechanisms of nutritional-induced delirium. Toward this aim, fluid and electrolyte balance disorders are encompassed within the descriptive process, considering the clinical context. Finally, clinical and research perspectives are addressed in a dedicated paragraph.

## 2. Causes of Malnutrition in the Critically Ill Patient

According to the European Society for Clinical Nutrition and Metabolism (ESPEN) definition, malnutrition is “a state resulting from lack of intake or uptake of nutrition that leads to altered body composition (decreased fat-free mass) and body cell mass leading to diminished physical and mental function and impaired clinical outcome from disease” [19]. It causes increased morbidity and mortality and has negative effects on the results of therapies [20]. Malnutrition is a common occurrence among patients, particularly the elderly, who suffer from chronic illnesses. This problem also impacts hospitalized patients. The documented prevalence of malnutrition in UK hospitals is up to 40%, with a significant number of patients experiencing a subsequent deterioration in their nutritional status throughout their hospital stay [21]. Up to 65% of patients undergoing hospital admission for surgical procedures demonstrate malnutrition or are at risk of nutritional deficiencies [22]. Notably, in oncological patients eligible for surgery, these values can increase. For instance, in individuals with gastric cancer, preoperative malnutrition can affect up to 85% of patients [23].

Concerning effects, malnutrition may reduce the immune response [24], increase intrahospital infections, delay healing, and compromise the function of organs and systems. In the ICU field, this condition induces ventilator dependence due to reduced muscle mass and strength [25]. It also predisposes to thromboembolism due to inactivity and induces depression and a lack of interest in food [26,27] or hospitalization, depending on the type of disease

**Figure 1:**
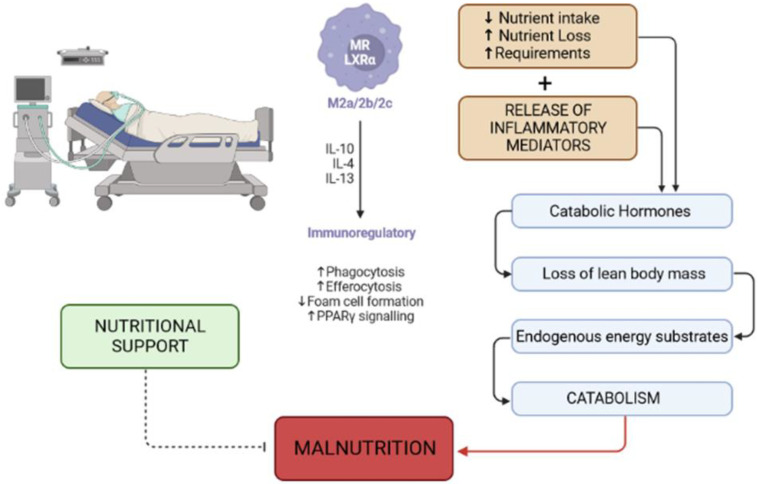
Causes and mechanisms of malnutrition in critically ill patients. Malnutrition can stem from various factors in critically ill patients. These factors may encompass reduced oral intake caused by digestive dysfunction and malabsorption, as well as increased nutrient loss resulting from conditions such as diarrhea, vomiting, or draining wounds. Sedatives or opioids can suppress appetite or impair nutrient absorption. Additionally, metabolic stress can trigger heightened energy and nutrient needs, while the inflammatory response can increase energy expenditure and protein breakdown and prompt complex metabolic alterations. Abbreviations: PPARs, Peroxisome Proliferator-Activated Receptors; MR, macrophage receptor; LXRα, Liver X Receptor alpha.

In critically ill patients, there are several potential causes of malnutrition. They may include reduced oral intake, and digestive dysfunction due to gastrointestinal problems, such as delayed gastric emptying or malabsorption with impaired nutrient absorption. Other causes of malnutrition include increased nutrient losses due to diarrhea, vomiting, or draining wounds; metabolic stress with increased energy and nutrient requirements, an inflammatory response that can lead to increased energy expenditure and protein breakdown; and medications that can suppress appetite or impair nutrient absorption such as sedatives or opioids. Finally, a wide range of acute or chronic diseases can affect nutritional status as well as pain and medication side-effects [27,28] (Figure 1).

In ICU patients, complex metabolic changes in macronutrients usually occur. There is insulin resistance and hyperglycemia [29] and an increased release of proinflammatory cytokines, which enhance the production of catabolic hormones (glucagon, catecholamines, and cortisol). These hormones stimulate glycogenolysis and gluconeogenesis in the liver to mobilize glucose for use by tissues and cells [30,31]. Glycogen stores are depleted in a short time and, therefore, endogenous fats and proteins become the main source of oxidative energy substrate [32].

Protein is the main energy substrate source during the catabolic stress phase of critical illness. The synthesis of acute-phase proteins and those involved in immune function increase to support recovery [33]. Consequently, there is a rapid and important loss of skeletal muscle mass to provide precursor amino acids to help this process [34]. In addition, stress hormones such as epinephrine, norepinephrine, and glucagon directly activate lipase, causing the breakdown of triglycerides stored in adipose tissue. This results in the release of free fatty acids (FFAs) and glycerol into the bloodstream [35]. More importantly, in critical illness, the condition of hypermetabolism can cause changes in energy expenditure [36]. In particular, it seems that hypermetabolism varies in the different phases of the disease and with the type of disease [37]. In this regard, studies have shown that resting energy expenditure (REE) is high from the first to the third week, even when sepsis or other causes of critical illness have been properly treated [38].

## 3. Nutritional Screening and Assessment

Initial nutritional screening should be performed within 48 h of admission to identify patients with malnutrition or at risk and determine whether the nutritional assessment is needed. The ESPEN guidelines suggest that every patient hospitalized in the ICU for more than 48 h should be considered malnourished [39]. Despite there being no validated and recommended tools to estimate the nutritional status of a critically ill patient, several tools to estimate nutritional risk can be used. They include the Nutrition Risk Score (NRS 2002), the NUTRIC (Nutrition Risk in the Critically Ill) Score, and the Subjective Global Assessment (EMS). These screenings are based on medical history (age, comorbidity, loss of physical function), nutritional history (weight loss, reduced food intake, loss of appetite), physical examination (body mass index, BMI, edema, body composition), and severity of the disease assessed by the SOFA (Sequential Organ Failure Assessment), the APACHE II (Acute Physiology and Chronic Health Evaluation), or other scores [20,40,41,42,43].

Furthermore, apart from nutritional scores, several technical tools, such as computed tomography (CT) analysis, musculoskeletal ultrasound, and bioelectrical impedance analysis (BIA), can be used for the assessment and monitoring of nutritional status in the ICU. Unfortunately, they are not widely implemented in the clinical routine [42].

### Bioelectrical Impedance Analysis

BIA is a non-invasive and simple method that allows for estimating body composition. BIA utilizes the electrically conductive properties of the human body by passing an alternating electric current through it [43]. In particular, BIA is based on impedance, which is a physical quantity that measures the opposition force to the flow of an alternating electric current by the tissues. Impedance is expressed by a combination of resistance and reactance, which constitute, respectively, the resistive and capacitive part of the impedance itself [44]. Resistance represents the opposition to the electric current through ionic solutions in intra- and extracellular cells and is inversely proportional to the water content of the tissue. Therefore, fat-free tissues are “good conductors” for fluid and electrolyte content which oppose, to the passage of current, a low resistance. Conversely, fat and bone tissues are “bad conductors” because they are poor in fluids and electrolytes. Reactance, on the other hand, constitutes the capacitive part of the impedance and is the force that a capacitor resists against the flow of a current. The cells of the organism behave like capacitors since the pair of conductors is represented by the intracellular fluids, while the dielectric insulator is represented by the non-conductive cell membrane [45] [Table 1].

Bioelectrical Impedance Vector Analysis (BIVA) is an integral part of BIA measurement. It is a simple, rapid, and clinically valid method for assessing fluid status (i.e., total body water, TBW) and body cell mass (BCM). This method plots the direct impedance resistance and reactance measurements as a bivariate vector in a nomogram [46]. BIVA has been reported to be useful for monitoring hydration status during fluid removal in patients with decompensated heart failure [47,48] and during intermittent hemodialysis [49,50]. Therefore, it can also prove useful in patients who are seriously ill.

## 4. Malnutrition and Delirium

There are significant challenges in interpreting studies on the correlations between malnutrition and delirium in critically ill patients, primarily due to multiple biases. The heterogeneity of methods used for diagnosing delirium and malnutrition makes it difficult to draw definitive conclusions. For example, commonly employed methods for diagnosing delirium in the ICU include the Confusion Assessment Method for the ICU (CAM-ICU), the Intensive Care Delirium Screening Checklist (ICDSC), and the CAM-ICU-7 (an extension of CAM-ICU, assessing a patient’s speech and language functions) [51]. Moreover, a combination of anthropometric measurements (e.g., BMI), biochemical markers (e.g., albumin), and tools such as the Malnutrition Universal Screening Tool (MUST) are used for identifying malnutrition [52]. Furthermore, in this clinical setting, there are several confounding factors that need to be considered. Undoubtedly, alterations in nutritional status and delirium have a strong association, with one condition often exacerbating the other. Malnutrition and low BMI values can result in several physiological changes that can contribute to the development of delirium, including dehydration [7,53,54,55,56,57,58], electrolyte imbalances [59,60,61,62], changes in drug metabolism [56,63], a deficiency of trace elements [55,63,64,65,66,67,68], metabolic alterations [17,69,70,71], as well as neuroinflammation and immune system dysfunction [72,73,74,75,76,77,78]. A special issue concerns the link between malnutrition, hemoglobin, and delirium.

### 4.1. Alterations in Body Fluid Composition

Dehydration is a common problem in critically ill patients in the ICU [53]. It can result from various factors, such as decreased fluid intake (for example, due to restricted fluid regimens), increased fluid output (due to factors like diuretic use, fluid loss from wounds or drains, fever, vomiting, diarrhea, or sweating, as well as medically induced diabetes insipidus), and hyperosmolar fluids like hypertonic saline or dextrose solutions, particularly when not adequately balanced with other fluids.

The alteration of body fluid composition has an important influence on the onset of delirium in patients undergoing elective surgery [54,56,57]. Nevertheless, the role of fluid deficit in the pathogenesis of delirium is not entirely clear. It probably involves tissue hypoperfusion, especially cerebral and renal, and an increased concentration of drugs and metabolites mostly due to their impaired renal clearance [57]. Therefore, these changes can interfere with cognitive functioning via impaired oxygen and glucose levels and low perfusion pressure in the brain [55,57]. Notably, these issues can be readily examined as the occurrence of dehydration can be addressed by careful monitoring of the volume status using cardiac output monitoring [79] or echocardiography [80,81].

Dehydration is thought to cause alterations in neuronal mitochondrial function with neuronal death, cytokine and nitrous oxide release, and neurotransmitter dysfunction, all of which may contribute to delirium. Several studies have shown that losses of up to 2% of total body water weight led to impaired visuospatial processing, an attenuation of short-term memory, and impaired performance of attention and psychomotor tasks [58,82].

### 4.2. Electrolyte Imbalances

Research also proved that electrolyte disturbances are closely related to postoperative delirium, but the influence of different electrolytes on postoperative and ICU delirium remains controversial [59,60,61]. Alterations in sodium or potassium levels affect body fluids, causing hypotonic or hyperosmotic dehydration. An altered potassium serum level may occur in association with metabolic alkalosis or microcirculation disorders, which may result in symptoms such as depression, apathy, fatigue, drowsiness, confusion, or coma [61].

In previous studies, the authors showed that lower serum levels of magnesium and phosphate were associated with postoperative delirium [83]. More recently, Wang et al. [62] found an association with calcium disorders, mostly hypocalcemia, and postoperative delirium occurrence. On the other hand, it is often difficult to establish a link between electrolyte disturbances and delirium, as serum levels of electrolytes such as potassium and magnesium do not always reflect total body content.

### 4.3. Changes in Drug Metabolism

Patients in the ICU may experience changes in drug metabolism due to a variety of factors, including organ dysfunction, changes in drug distribution and elimination, and drug–drug interactions. Organ dysfunction, such as renal or hepatic failure, can compromise the capacity to metabolize drugs, leading to decreased clearance and increased drug levels in the blood. In addition, alterations in drug distribution and elimination may also occur in critically ill patients due to shifts in body fluids, changes in plasma protein levels, and alterations in drug absorption. Furthermore, critically ill patients are often on multiple medications, which can increase the risk of drug–drug interactions. These interactions can lead to changes in drug metabolism, as well as potential adverse effects such as toxicity or reduced efficacy [84].

Malnutrition can have a significant impact on drug metabolism, especially in critically ill patients. Consequently, it can induce delirium through pharmacokinetic (PK) alterations of deliriogenic drugs such as benzodiazepines. In particular, malnutrition can cause various modifications in the body, such as decreased liver and kidney function, reduced blood flow to organs, and an altered absorption and distribution of drugs. As a result, malnutrition can affect drug metabolism by reducing the activity of liver enzymes involved in drug metabolism, leading to a longer half-life and increased toxicity of some drugs. Additionally, malnutrition can also cause changes in drug absorption and clearance.

Malnourished patients may also have an altered protein binding of drugs, leading to increased levels of the free (unbound) drug in the blood [56]. This can result in increased toxicity, as many drugs are bound to proteins in the blood and only the unbound fraction is available for metabolism and elimination.

It is difficult to establish the role of malnutrition in the development of PK-induced delirium in the ICU. In critically ill patients, indeed, malnutrition is often accompanied by other factors that can further affect drug metabolism, such as organ dysfunction, inflammation, and drug–drug interactions [63].

### 4.4. Deficiency of Trace Elements

The deficiency of trace elements has been associated with the development of delirium. For example, vitamin deficiencies, such as a lack of niacin or thiamine, have been shown to affect the development of delirium by disrupting normal neurotransmission [55]. Furthermore, cobalamin (vitamin B12) deficiency is a prevalent factor that can lead to the manifestation of various neuropsychiatric symptoms. In elderly patients undergoing cardiac surgery, the incidence of delirium was significantly higher in patients with cobalamin deficiency (42%) compared to those without (26%) (*p* = 0.017). Furthermore, the severity of delirium was also significantly greater in patients with cobalamin deficiency (16.5 ± 2.9) in contrast to patients without cobalamin deficiency (15.03 ± 2.48) (*p* = 0.034) [64]. In a prospective cohort study conducted in the setting of cardiac surgery (*n =* 296), Shariatpanahi et al. [64] demonstrated that folate deficiency and hyperhomocysteinemia were observed in 6% and 68% of patients, respectively, although the findings were not significant. Additionally, a recent meta-analysis found that vitamin D insufficiency (indicated by serum values of 20–29 ng/mL or 50 nmol/L), but not vitamin D deficiency (f < 20 ng/mL), was significantly associated with the development of postoperative delirium, with a *p*-value less than 0.01 [66]. Moreover, low levels of vitamin D are also associated with dysregulated inorganic phosphate metabolism, and abnormal levels of serum phosphate should also be investigated [85].

The data on vitamin C are controversial and require further investigation. In a retrospective study, the administration of vitamin C (3 g/12 h or 1.5 g/6 h plus thiamine 200 mg/12 h) in septic patients showed no association with ICU delirium-free days among patients in septic shock [67]. On the contrary, in an early investigation, Voigt et al. [68] measured the concentration of vitamin C in the plasma and cerebrospinal fluid (CSF) of patients with septic encephalopathy. They found a significant decrease in vitamin C levels in both the plasma and CSF of patients with septic encephalopathy compared to controls. Additionally, they observed that the degree of decrease in vitamin C concentration in the CSF was associated with the severity of neurological symptoms. It is important to note that since vitamin C is an acute-phase reactant, its levels change in inflammatory conditions [86].

### 4.5. Metabolic Alterations

Both high and low glucose levels can be associated with an increased risk of delirium in critically ill patients, particularly those with pre-existing cognitive impairment or neurological conditions. Severe hypoglycemia causes a reduction in intracellular neuronal ATP levels, which, downstream, leads to neuronal hyperpolarization and alterations in hippocampal intrinsic rhythms [69]. Clinically, it results in seizures and decreased cognition, both risk factors for delirium. Similarly, hyperglycemia, both acute and chronic, has been shown to induce oxidative stress, neuroinflammation, and neuronal damage, with cognitive decline. In particular, patients with diabetes mellitus are at least one-and-a-half times more likely to develop dementia than individuals without diabetes [70]. It demonstrates the role and long-term consequences of alterations in glucose on the functioning of the brain.

Diverse studies have focused on the link between albumin levels and delirium. A meta-analysis of risk factors for the onset of delirium among elderly patients in the medical ICU showed that a low albumin level is associated with delirium [17]. Zhang et al. [71] studied 700 elderly patients admitted to the ICU after noncardiac surgery and observed that preoperative hypoalbuminemia was associated with an increased risk of postoperative delirium. Albumin is an acute-phase reactant associated with inflammation [87].

### 4.6. Neuroinflammation

Neuroinflammation is an interesting field of study for investigating the link between malnutrition and delirium in the ICU. Systemic inflammatory processes may affect brain function through microglial activation and blood–brain barrier (BBB) alterations [72]. In this complex scenario, peripheral cytokines could act through neurodegeneration, or indirectly, with effects on neurotransmission changes. Several studies have shown that peripheral inflammatory stimuli induce a profound immunological response in the brain through the activation of microglia [73]. In particular, cytokine dysregulation can cause neuronal injury through several mechanisms, such as impaired neurotransmission, apoptosis, and the activation of microglia and astrocytes, leading to the production of glutamate, free radicals, complement factors, and nitric oxide [74]. Systemic inflammation activates vascular endothelial and perivascular cells located close to the BBB, propagating the inflammatory cascade and directly or indirectly damaging neurons [75].

Several pathways of this cascade have been investigated. For example, Heimberger et al. [76] recently demonstrated that the presence of pro-inflammatory cytokines triggers kynurenine pathway activation with metabolite accumulation, immunosuppression, and neuroactive and pro-oxidative activities, potentially with a direct effect on neuronal and microglial activities.

Inflammatory alterations are responsible for the various clinical manifestations of delirium. Different proofs have linked elevated levels of the interleukins IL-6 and IL-8 to delirium. It was noted that IL-6 was specifically associated with the hyperactive form of delirium, whereas IL-8 was highest in the days leading up to the onset of delirium. Moreover, elevated IL-6 in association with delirium was similarly found in a study of elderly patients after abdominal surgery [77].

**Figure 2:**
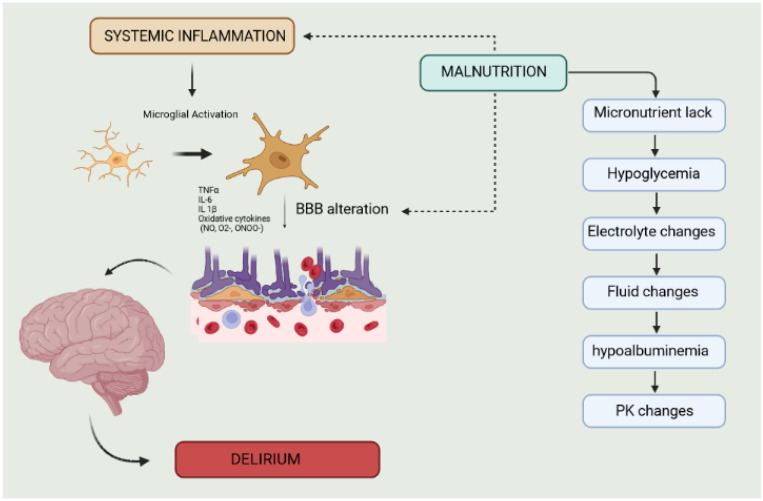
The link between malnutrition and delirium. The development of malnutrition-associated delirium is a complex process, involving multiple interrelated pathways. Metabolic factors can lead to pharmacokinetic (PK) changes and can also trigger proinflammatory responses. This can result in the accumulation of toxic metabolites and elevated levels of inflammatory cytokines, which can further exacerbate the problem. Alteration of the blood–brain barrier (BBB) and neuroinflammation cause result in neurotoxicity. Other factors such as anemia due to nutrient deficiencies (e.g., iron, vitamin B12, and folate) and decreased hemoglobin production, and impaired hematopoiesis concur.

The pathogenesis of malnutrition-associated delirium is very intricate. The cascades are likely to overlap. Therefore, metabolic factors can induce PK alterations, but also induce proinflammatory processes, in turn, supported by the toxicity of metabolites and the increased level of inflammatory cytokines. For example, low albumin is associated with a high number of free drugs and toxic metabolites. They impact an injured BBB due to neuroinflammation, promoting brain absorption and neurotoxicity [78]. Furthermore, inflammatory activation in critically ill patients can lead to additional metabolic alterations, such as a decline in serum vitamin D concentrations and dysregulated phosphate [66] (Figure 2).

### 4.7. Malnutrition and Anemia

Malnutrition and hemoglobin (Hb) levels are interconnected, and their relationship can have significant implications for the development of delirium. For example, malnutrition can induce nutrient deficiencies, including iron, vitamin B12, and folate. These nutrients are essential for the synthesis of hemoglobin. In the absence of an adequate supply of these nutrients, the production of hemoglobin may be impaired, leading to a decrease in hemoglobin levels. Moreover, malnutrition can negatively impact hematopoiesis and contribute to chronic inflammatory conditions that affect the regulation of iron metabolism [88]. Therefore, following this deleterious cascade, hemoglobin levels may be decreased despite the presence of adequate iron stores [89].

Hb changes have been implicated in the pathophysiology of delirium. Although the exact mechanisms linking hemoglobin levels and delirium are not fully understood, several factors contribute to this association [90]. These factors include a reduced oxygen supply to the brain and the impact on inflammatory processes [91]. In a recent retrospective analysis, the authors underscored a notable link between diminished pre-operative Hb concentrations and significant fluctuations in Hb levels, indicating an elevated risk of postoperative delirium. Furthermore, factors such as advanced age, a history of stroke, transfer to the ICU postoperatively, and experiencing pain within the initial 2 days following surgery exhibited noteworthy associations in this study [92].

## 5. Clinical and Research Perspectives

### 5.1. Clinical Perspectives

The concept that poor nutritional status may be associated with postoperative delirium is a significant finding that suggests a potential continuum between varying degrees of malnutrition and the risk of developing this acute complication. Therefore, it is crucial that all patients undergo nutritional assessment upon admission to the hospital to identify those who may be at risk.

To effectively manage malnutrition and prevent delirium in critically ill patients, a multidisciplinary approach involving a nutritionist, physician, and other health professionals may be necessary [93]. In this context, greater attention to nutritional assessment with tools and BIA can lead to more accurate and comprehensive evaluations of a patient’s nutritional status. However, despite these diagnostic strategies being simple and cost-effective, research on the use of these approaches for delirium prevention is lacking.

Additionally, early treatment of hydroelectrolytic [94] and nutritional disorders should be prioritized to prevent the development of delirium. In this regard, sufficient fluid intake or fluid supplementation and adequate nutrition with feeding assistance have been recognized as key elements among the multimodal non-pharmacological preventive strategies [95].

### 5.2. Research Perspectives

Evidence-based medicine analyses could offer valuable information on different topics. According to a network meta-analysis, paying attention to nutrition and hydration may be linked to a decrease in the incidence of delirium (OR 0.48, 95% CrI 0.18 to 1.26). However, the estimates also accounted for the potential lack of benefit [96]. This finding highlights the need to further develop research in this area (Table 2).

Research should be directed toward investigating the links between nutritional factors and multiple phenotypic features of delirium, such as its onset, duration, and clinical manifestations (subtypes). For example, the impact of early enteral feeding on the incidence and severity of clinical manifestations could be investigated through the analysis of large datasets.

Clinical trials can be used for investigating delirium in at-risk patients and in specific populations, for example, those with sepsis, traumatic brain injury, or acute respiratory distress syndrome. In an earlier study, Ringaitiene et al. [97] established a connection between postoperative delirium and malnutrition in patients who underwent on-pump coronary artery bypass grafting. Similarly, a separate investigation found a correlation between preoperative malnutrition and postoperative delirium among older patients undergoing hip fracture surgery [98].

Concerning predictive biomarkers, at present, there is no substantiated evidence for the application of a specific biomarker [99]. Nevertheless, target metabolomic analysis shows interesting promise. For example, Tripp et al. [100] demonstrated that disruptions in energy metabolism and amino acid synthesis pathways are strictly linked to postoperative delirium, indicating possible mechanisms underlying the development of this condition. Remarkably, it was demonstrated that some proteins are important before surgery and could be investigated as risk markers; on the contrary, others increase their serum concentrations at the time of delirium and can be used as disease markers [101]. Therefore, metabolomic analyses could be useful for establishing dynamic signatures of ICU delirium.

Preclinical research has a key role in this complicated field. Despite the fact that the use of animal models to study delirium is still in its early stages, there is a pressing need for meticulous and high-quality research in this field to improve our comprehension of its intricate neurobiology [102]. Once the most accurate preclinical models are established, this research could test the role of nutritional strategies in delirium prevention and the link between delirium and nutrition.

Finally, machine learning (ML) and artificial intelligence (AI) strategies represent a valuable opportunity for research. AI-based approaches may be particularly promising for filling research gaps, enabling broader analyses and the more efficient identification of emerging trends. For example, ML algorithms can be a strong opportunity to analyze patient histories, medications, and environmental factors to identify potential triggers for delirium. This can guide targeted interventions and preventative measures. Moreover, natural language processing (NLP) (an AI subfield) algorithms can be used to analyze a vast amount of literature, extracting insights related to delirium and nutrition. This aids in staying updated with the latest research and informs future studies. Furthermore, deep learning approaches can be adopted to develop models that continuously analyze real-time data, triggering alerts when abnormal patterns are detected. These automated surveillance systems can be useful to ensure timely responses and interventions (Table 3).

## 6. Conclusions

The underlying mechanisms of the relationship between malnutrition and delirium are not yet fully understood. However, it is believed that malnutrition may lead to changes in brain chemistry and function, which can contribute to the development of delirium. Malnutrition can also weaken the immune system, making patients more susceptible to infections and inflammation, which are known risk factors for delirium. Studies have shown that addressing poor nutritional status in patients can help to prevent or reduce the severity of delirium. In some cases, nutritional interventions, such as providing adequate protein and caloric intake, can even help to reverse the effects of delirium. Therefore, identifying and addressing poor nutritional status in patients may be an important strategy for preventing and managing delirium in clinical settings.

## Figures and Tables

**Table 1:** Bioelectrical impedance analysis parameters.

Parameter	Notes	Findings
BCM	All cells that influence metabolism (e.g., muscle, internal organs, nervous system)	↑ good training status, intracellular water retention
		↓ malnutrition, cachexia, dehydration
BCM %	The percentage of BCM in FFM which measures individual nutritional status and physical fitness level	↑good training status
		↓ malnutrition
EC	Mainly extracellular water	↑ extracellular water retention (e.g., edema)
		↓ extracellular loss of water (e.g., diuretics)
FFM	No body fat tissues. It consists of BCM and ECM	↓ elderly, chronic diseases
FM	It is indirectly determined from body weight minus FFM	
Phase angle	Key indicator of cell membrane function	
TBW		↑ high portion of the muscle, water retention (e.g., edema)
		↓ small portion of muscle, dehydration/insufficient fluid intake

Abbreviations: body cell mass (BCM), extracellular mass (ECM), fat-free mass (FFM), fat mass (FM), total body water (TBW).

**Table 2:** Suggestions for research on nutrition and delirium in the ICU.

Research Topic	Proposed Strategy(ies)
The impact of early enteral feeding on the incidence and severity of clinical manifestations.	Analysis of large datasets through AI methods.
Efficacy of nutritional supplementation in preventing delirium in at-risk patients.	AI-based strategies; multicentric investigations; EBM studies.
Investigations in specific patient populations.	For example, those with sepsis, traumatic brain injury, or acute respiratory distress syndrome.
Gender differences and age groups	Retrospective cohort studies to examine the association between pre-existing conditions, medications, and the incidence of delirium in male and female patients across different age categories. Meta-analyses and systematic reviews. AI-based strategies.
Relationship between specific nutrients and the development of delirium in critically ill patients.	For example, vitamin B12 or omega-3 fatty acids.
Potential benefits of parenteral nutrition on delirium incidence in ICU patients who are unable to tolerate enteral feeding.	Clinical trial (e.g., prospective investigations).
The role of individualized nutrition plans on delirium prevention and management.	Clinical trial (e.g., prospective investigations). Nutritional assessment (e.g., BIA)
The potential association between delirium and markers of malnutrition.	Serum albumin levels or prealbumin.
The impact of different routes of feeding (enteral vs. parenteral) on the development of delirium.	Retrospective analyses on large datasets.
The role of nutritional interventions in reducing the duration of delirium.	AI studies; EBM analyses; clinical trials
The impact of regular hydration and electrolyte management on the incidence and severity of delirium.	AI studies; EBM analyses; clinical trials
Assessment of the impact of nutritional interventions on cognitive outcomes in those who have experienced delirium.	Long-time follow-up studies.
Multiprofessional approaches.	Nurse-led nutritional interventions in preventing delirium and improving outcomes.
Predictive biomarkers.	Target metabolomic analysis.
Preclinical research.	Development of reliable animal models.

Abbreviations: artificial intelligence (AI); bioelectrical impedance analysis (BIA); evidence-based medicine (EBM); intensive care unit (ICU).

**Table 3:** Potential artificial intelligence strategies for studying delirium and nutrition in ICU.

Artificial Intelligence Approach	Applications	Aims
Machine learning (ML) algorithms	Development of predictive models on datasets comprising patient demographics, medical history, medications, and vital signs.	Delirium risk (e.g., phenotypes)
ML algorithms	Predictive ML-based models on datasets including patient data, nutritional history, metabolic rates, and response to interventions.	Tailored nutritional plans, optimizing recovery, and minimizing complications
Computer vision (CV) and ML	Use of CV for analyzing patient images to estimate body composition changes, and ML for processing nutritional intake records, lab values, and patient-reported outcomes.	To assess and predict nutritional status.
Deep learning approaches	Development of models that continuously analyze real-time data, triggering alerts when abnormal patterns are detected.	Automated surveillance systems can be useful to ensure timely responses and interventions.
Natural language processing	Analysis of a vast amount of literature for extracting insights.	Extensive literature review.

## Data Availability

The datasets used and analyzed for the current review are available from the corresponding author upon reasonable request.
